# Transoral Robotic Surgery for Elderly Patients with Oropharyngeal and Laryngeal Cancer: A Comprehensive Review

**DOI:** 10.3390/jcm15041586

**Published:** 2026-02-18

**Authors:** Elena Russo, David Virós Porcuna, Philippe Gorphe, Vinidh Paleri, Raul Pellini, Andrea Costantino, Remo Accorona, Armando De Virgilio

**Affiliations:** 1 Department of Organs of Sense, ‘Sapienza’ University of Rome, 00161 Rome, Italy; armando.devirgilio@gmail.com; 2 Otolaryngology Section, Head and Neck Surgery, Hospital Germans Trias I Pujol, 08916 Badalona, Spain; david.viros@vhir.org; 3 Department of Head and Neck Oncology, Gustave Roussy Cancer Campus, 94800 Villjuif, France; philippe.gorphe@gustaveroussy.fr; 4 Royal Marsden Hospital, London SW3 6JJ, UK; vinidh.paleri@rmh.nhs.uk; 5 Department of Otolaryngology—Head & Neck Surgery, IRCCS Regina Elena National Cancer Institute, Via Elio Chianesi 53, 00128 Rome, Italy; raul.pellini@ifo.it; 6 Otorhinolaryngology Unit, IRCCS Humanitas Research Hospital, Via Manzoni 56, Rozzano, 20089 Milan, Italy; andrea.costantino94@gmail.com; 7 Department of Otolaryngology—Head and Neck Surgery, Niguarda Hospital, 20162 Milan, Italy; remo.accorona@icloud.com

**Keywords:** head and neck cancer, transoral robotic surgery, elderly patients, age, survival, functional outcomes

## Abstract

**Background/Objectives**: Head and neck squamous cell carcinoma (HNSCC) poses a significant health challenge, especially among elderly patients, who are often underrepresented in clinical trials. Transoral robotic surgery (TORS) has emerged as a promising alternative to non-surgical strategies such as chemoradiotherapy (CRT), but its effectiveness in older adults is not well-studied. **Methods**: A structured narrative review of studies on TORS for elderly HNSCC patients was conducted using the PubMed/MEDLINE database. Studies were selected according to predefined eligibility criteria based on the PICOS framework. PRISMA reporting principles were applied to document study identification and selection. **Results**: The available evidence suggests that, in carefully selected elderly patients, TORS is associated with disease-specific (DSS) and disease-free survival (DFS) outcomes comparable to those reported in younger cohorts, while overall survival (OS) appears more strongly influenced by comorbidities than chronological age. TORS may facilitate treatment de-escalation in selected cases, potentially reducing exposure to adjuvant therapies and limiting treatment-related toxicity. Functional outcomes, particularly swallowing function and long-term gastrostomy dependence, may be favorable in selected elderly patients; however, comparative data with non-surgical approaches remain limited, heterogeneous, and are partly derived from mixed-age cohorts. **Conclusions**: TORS represents a viable treatment option for selected elderly HNSCC patients, providing encouraging oncologic outcomes and potential functional advantages. Nevertheless, the current evidence base is predominantly retrospective and heterogeneous. Careful patient selection is essential, and further prospective elderly-specific studies are needed to better define functional and oncologic benefits.

## 1. Introduction

Head and neck squamous cell carcinoma (HNSCC) is the sixth most common cancer globally, accounting for 5.3% of all cancer cases [1]. Approximately 30% of HNSCC diagnoses occur in patients over the age of 70 [2]. With the increasing life expectancy and ongoing population growth, the cancer burden in the elderly is on the rise. Additionally, one of the fastest-growing segments of HNSCC cases is among patients with human papillomavirus (HPV)-related HNSCC. Although HPV-positive patients are typically younger, often in their fourth or fifth decade of life, and with fewer comorbidities, there is a growing cohort of elderly individuals affected by HPV-mediated HNSCC [3,4,5,6]. In the United States, one-third of HPV-positive HNSCC patients are older than 65 years [4].

Treatment strategies for HNSCC involve upfront surgery, which is often followed by adjuvant radiotherapy (RT) or concurrent chemoradiotherapy (CRT), depending on the tumor’s pathologic characteristics. Alternatively, some patients may be treated exclusively with RT or CRT. However, there is currently a notable absence of adequate evidence-based guidelines specifically addressing the treatment of older adults with HNSCC. Several factors contribute to this gap. First, elderly patients are often underrepresented in clinical trials, accounting for less than 5% of enrolled participants [7]. This underrepresentation undermines the validity of findings for this population. Second, many elderly patients face a higher burden of comorbidities, which can reduce their tolerance for multimodal therapies, especially those including chemotherapy, due to an increased risk of side effects [7,8,9]. Age-related physiological changes, such as sarcopenia, decreased lung function, cognitive decline, and weakened immune function, further elevate the risk of delayed recovery, unexpected hospitalizations, and treatment interruptions in older patients [10,11]. Third, research has shown that older age is the most significant predictor of severe late toxicities following CRT [12]. Moreover, several subgroup analyses from chemotherapy trials indicate that there is no survival benefit for patients over 70 years old. This lack of benefit is likely due to the toxic effects of cisplatin outweighing its potential advantages in treating cancer [13,14]. Additionally, elderly patients often have a reduced ability to tolerate postoperative complications and face a higher risk of significant, potentially irreversible declines in their quality of life if they experience severe adverse events. These factors highlight the importance of personalized treatment plans, which should be based on a comprehensive geriatric assessment that aims to minimize toxicity while ensuring efficacy for this vulnerable population [15].

Over the past decade, transoral robotic surgery (TORS) has emerged as an alternative to definitive concurrent CRT, often in conjunction with adjuvant RT [16,17,18,19]. Since the Food and Drug Administration (FDA) approved TORS for treating early-stage oropharyngeal squamous cell carcinomas, it has transformed the surgical approach to oropharyngeal cancer [19,20]. This transformation can be attributed to TORS’s ability to provide excellent visualization of the anatomy without the need for more invasive procedures such as mandibulotomy or pharyngotomy to access tumors. In recent years, TORS has also gained traction as an alternative to transoral laser microsurgery for the treatment of selected laryngeal and hypopharyngeal tumors [21,22,23,24]. Numerous studies have reported outstanding outcomes with TORS concerning both survival and functional status [18,19,20,21,22,23,24]. However, despite these positive results, elderly patients are often less likely to receive surgical treatment than their younger counterparts, likely due to concerns about their perceived frailty [4]. There is limited data on the functional and oncologic outcomes of TORS, specifically in the elderly population. Thus, this study aims to review the indications, contraindications, and therapeutic outcomes of TORS in elderly patients affected by HNSCC.

## 2. Materials and Methods

### 2.1. Study Design and Literature Research Strategy

This study was conducted as a structured narrative review aimed at providing a comprehensive, clinically oriented synthesis of the available evidence on TORS in elderly patients with head and neck cancer.

To enhance transparency in study identification, a systematic literature search strategy was implemented. Selected reporting principles from the Preferred Reporting Items for Systematic Reviews and Meta-Analyses (PRISMA) statement were followed where applicable, particularly regarding search strategy description and study selection. However, given the narrative design of the review, PRISMA items relating to quantitative synthesis, protocol registration, and formal risk-of-bias scoring were not applied.

A comprehensive literature search was performed in the PubMed/MEDLINE database. The full search strategy was: (‘Robotic Surgical Procedures’[MeSH Terms] OR ‘robotic surgery’[All Fields] OR ‘transoral robotic surgery’[All Fields] OR ‘TORS’[All Fields] OR ‘robotics’[All Fields]) AND (‘aged’[MeSH Terms] OR ‘elderly patient’[All Fields] OR ‘older adult’[All Fields] OR ‘geriatric patient’[All Fields] OR ‘aging patient’[All Fields] OR ‘over 70’[All Fields]). Given the narrative design of this review, additional bibliographic databases were not systematically searched. PubMed/MEDLINE was selected as the primary source due to its comprehensive coverage of biomedical and head and neck oncologic literature. In addition, an extensive manual search of the references from all relevant studies was performed. The last search was conducted on 31 March 2025. Ethics committee approval was not required for this review, as it was based exclusively on previously published literature.

### 2.2. Eligibility Criteria

Study eligibility was defined using the PICOS framework to ensure consistency in study identification and selection: Patients (P), elderly patients (defined as age ≥ 65 years) diagnosed with head and neck squamous cell carcinoma; Intervention (I), TORS, either as a primary treatment or as part of a multimodal treatment strategy; Comparator (C), RT/CRT, other surgical approaches (including transoral laser microsurgery), or no comparator; Outcomes (O), oncologic outcomes (overall survival, disease-specific survival, disease-free survival, local or regional control) and/or functional outcomes (swallowing function, gastrostomy dependence, tracheostomy rates, quality-of-life measures); Study design (S), clinical studies, including prospective and retrospective observational studies, and clinical trials.

The ≥65-year threshold was selected in accordance with the World Health Organization and commonly adopted oncogeriatric definitions of older adulthood. However, we acknowledge that age cutoffs vary across the head and neck oncology literature, with many surgical series defining elderly cohorts as ≥70 years or older. Accordingly, age definitions were extracted and reported for each included study, and findings were interpreted in the context of this heterogeneity.

Exclusion criteria were non-clinical studies (technical reports, cadaveric or animal studies); case reports or small case series including fewer than five patients; conference abstracts without full-text availability; studies not reporting age-specific data relevant to elderly patients; and non-English language publications.

### 2.3. Study Selection

All retrieved records were screened by title and abstract for relevance. Full texts of potentially eligible studies were then assessed for inclusion according to predefined criteria. Any additional relevant studies identified through manual reference screening were also evaluated. Studies that met strict PICOS eligibility criteria were included in the primary qualitative synthesis. Given the narrative scope of this review, selected additional studies that did not meet all PICOS criteria were cited narratively to provide clinical context, support interpretation of findings, and summarize broader evidence relevant to transoral robotic surgery in elderly patients with head and neck cancer.

### 2.4. Quality Assessment

Given the heterogeneity of study designs and the predominance of retrospective observational studies, a formal quantitative risk-of-bias assessment using structured scoring tools was not performed. This decision was made in light of the narrative and interpretative design of the review and the absence of pooled outcome analyses. Instead, study quality was appraised using a structured qualitative framework. The following domains were systematically considered for each included study: study design, sample size and population representativeness, definition of the elderly cohort, clarity and completeness of oncologic and/or functional outcome reporting, duration and adequacy of follow-up, and potential risks of selection, treatment allocation, and reporting bias. This qualitative appraisal informed the interpretation of findings and the weighting of evidence in the narrative synthesis. Key methodological characteristics and limitations of the included studies are summarized in Table 1.

## 3. Results

### Search Results and Studies Description

The literature search identified 12,578 records through PubMed, with an additional 41 records retrieved through citation searching. After removal of 648 duplicates, 11,930 titles were screened, leading to the exclusion of 11,720 records.

A total of 212 reports were sought for retrieval (210 from database searching and 2 from citation tracking), of which 3 were not retrieved. Consequently, 104 full-text articles were assessed for eligibility. Following application of the predefined inclusion criteria, 98 reports were excluded.

**Figure 1:**
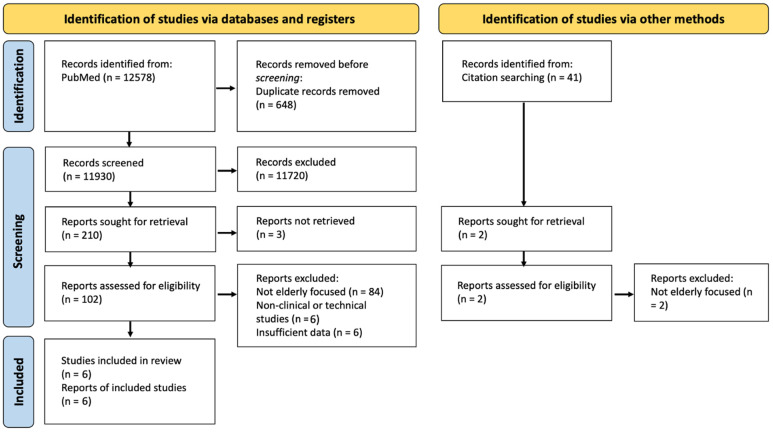
PRISMA flow diagram.

Ultimately, 6 studies were included in the narrative synthesis [25,26,27,28,29,30,31]. Figure 1 illustrates the study identification process and reasons for exclusion, while Table 1 summarizes the general characteristics of the included studies.

**Table 1:** General characteristics of the included studies. Abbreviations: FU follow-up; OPSCC oropharyngeal squamous cell carcinoma; TOS transoral surgery; TORS transoral robotic surgery; (C)RT (chemo)radiotherapy; DSS disease-specific survival; DFS disease-free survival; OS overall survival; FOIS functional oral intake scale; OR odds ratio; HR hazard ratio.

Author (Year)	Study Design	Population	Sample Size (Males)	Mean Age (y) (Range)	Median FU (m) (Range)	Key Findings	Methodological Considerations
Jackson et al. (2019) [25]	Retrospective case series	Patients ≥ 70 years old with HPV-positive OPSCC undergoing TOS	75 (67)	74 (70–87)	35.7 (1–167)	3-y DSS 94.3%, DFS 79.3%, OS 81.5%; survival comparable to younger cohorts; OS impacted by comorbidity-related mortality; prognostic stratification showed OS declining from 100% to 54% based on age, ACE-27 score, and cT stage	Retrospective design; single disease subtype (HPV + OPSCC); limited generalizability to non-HPV tumors; comorbidity confounding possible.
Parhar et al. (2020) [26]	Retrospective case series	Patients ≥ 70 years old with HPV-positive OPSCC undergoing TORS	77 (58)	73 (70–89)	39.6 (0.1–96.2)	3-y DSS 92.4%, OS 90.0%, DFS 84.3%; perioperative mortality 1.3% and postoperative hemorrhage 2.6% (below commonly reported TORS rates); no significant association between advanced comorbidity and OS or DFS	Retrospective series; perioperative outcomes well reported; limited comparative analysis; selection bias possible.
Costantino et al. (2025) [27]	Retrospective cohort study	Patients ≥ 65 years old with OPSCC undergoing TOS and adjuvant (C)RT	998 (816)	70 (N/A)	55 (N/A)	Risk-stratified cohort (low 34.8%, intermediate 26.1%, high 39.1%); no survival benefit from adjuvant therapy in low- or intermediate-risk disease; significant survival improvement in high-risk patients (HR 0.40); CRT marginally superior to RT alone; findings support risk-adapted adjuvant treatment selection in elderly OPSCC	Large cohort; risk-stratified analysis; retrospective registry design; treatment allocation bias possible.
Philips et al. (2020) [28]	Retrospective cohort study	Patients aged ≥ 70 years versus <70 years with OPSCC undergoing TORS	246 (203)	63.5 (N/A)	N/A	Higher discharge enteric access in elderly patients (*p* < 0.002); no difference by 3 months in enteral feeding requirement, diet, or FOIS scores; tracheostomy and unplanned readmission rates comparable between age groups; no significant OS or DFS differences when stratified by p16 status	Age-comparative cohort; functional outcomes included; short-term follow-up emphasis.
Costantino et al. (2024) [29]	Retrospective cohort study	Patients aged ≥ 70 years versus <70 years with OPSCC undergoing TOS	10,430 (8744)	60.7 (N/A)	N/A	Postoperative mortality comparable at 30 and 90 days; length of stay and 30-day readmission rates comparable between age groups; elderly patients less likely to receive indicated adjuvant RT (OR 0.69) or CT (OR 0.63), often due to refusal or comorbidity	Very large database study; strong statistical power; limited granularity on functional outcomes and frailty metrics.
Costantino et al. (2025) [30]	Retrospective cohort study	Patients aged ≥ 70 years with HPV-positive OPSCC undergoing TOS	2566 (2104)	N/A	48.2 (N/A)	Higher 5-year OS with primary TOS (81.6%) compared to primary RT (70.7%); propensity-matched analysis confirmed survival advantage for TOS (HR 0.64; *p* < 0.001)	Propensity-matched analysis; retrospective design; residual confounding cannot be excluded.

## 4. Discussion

### 4.1. TORS for Oropharyngeal Squamous Cell Carcinoma (OPSCC) in Elderly Patients

Since the FDA approved TORS for treating early-stage oropharyngeal squamous cell carcinomas (OPSCC), the surgical approach to oropharyngeal cancer, both HPV-related and non-HPV-related, has been significantly transformed, with important prognostic and therapeutic differences between these two disease entities [19,20]. However, there are only a few studies focusing on outcomes in elderly patients with HPV-related OPSCC [31]. It is important to note that the definition of “elderly” varies across these studies, most commonly using thresholds of ≥70 years, although some include broader age ranges. As a result, direct cross-study comparisons must be interpreted with caution, and oncologic outcomes should be considered within the context of differing chronological and physiological age profiles.

Jackson et al. [25] conducted a study evaluating oncologic outcomes in a cohort of 75 patients aged 70 years or older with HPV-related OPSCC who underwent transoral surgery, either TORS or transoral laser microsurgery (TOLM). Their findings indicated that elderly patients had comparable three-year disease-specific survival (DSS, 94.3%) and disease-free survival (DFS, 79.3%) to published data on younger cohorts [32,33]. However, overall survival (OS, 81.5%) was slightly lower than the typical 83–90% reported for HPV-associated OPSCC, primarily due to 21.3% of patients dying of unrelated causes [33,34,35,36]. Beyond chronological age alone, the study identified clinically meaningful prognostic stratification within the elderly population. Patients were distributed across three age groups (70–74, 75–79, and ≥80 years), and survival analyses demonstrated a clear gradient in outcomes when age was considered alongside comorbidity burden and tumor stage. On multivariable analysis, severe comorbidity as measured by the Adult Comorbidity Evaluation-27 (ACE-27) index and advanced clinical T stage were independently associated with worse DFS and OS. Through conjunctive consolidation modeling, three prognostic subgroups were defined, showing a marked decline in three-year OS from 100% in the most favorable category to 54% in the highest-risk group. These findings highlight the combined impact of physiologic vulnerability and disease burden on survival in elderly surgical candidates. However, the study did not clarify how many patients underwent TORS versus TOLM, despite the differences between these techniques in terms of advantages and limitations. Expanding on this research, Parhar et al. [26] analyzed a cohort of 77 patients aged 70 or older with HPV-related OPSCC who underwent primary TORS, with or without adjuvant RT. Their results showed three-year survival rates of 92.4% for DSS, 90.0% for OS, and 84.3% for DFS. The treatment approach demonstrated a favorable safety profile, with a perioperative mortality rate of 1.3% and a postoperative oropharyngeal hemorrhage rate of 2.6%, which compared well with reported hemorrhage rates after TORS (4.0–5.8%) [37,38]. Unlike Jackson et al. [25], they did not find a significant association between advanced comorbidities and poorer OS or DFS. Collectively, these findings reinforce the favorable oncologic profile of HPV-related OPSCC in elderly patients undergoing transoral surgery, with survival outcomes comparable to those reported in younger HPV-positive cohorts. In contrast, data specifically addressing HPV-negative elderly patients treated with TORS remain limited and are frequently embedded within mixed HPV-status cohorts, precluding robust subgroup efficacy analyses.

Head-to-head comparisons between primary TORS and primary RT for OPSCC are generally lacking. The ORATOR trial did not show a significant difference between the two approaches, but it was underpowered (34 patients per arm) [39]. NCDB analyses in older patients suggested that primary transoral surgery may be associated with better survival than primary RT, but the lack of data on recurrences and functional outcomes limits interpretation. Given the sample size required, a prospective study of this magnitude is unlikely to be feasible, especially in the elderly [30].

Notably, their findings align with previous research examining survival outcomes in older adults with HPV-associated OPSCC undergoing primary CRT. For instance, Hanasoge et al. [40] reported on 21 patients aged 70 or older who underwent definitive CRT for HPV-related OPSCC, finding a five-year OS rate of 76% with a median follow-up of 22.4 months. Similarly, Zumsteg et al. [41] analyzed 74 elderly patients treated with definitive CRT, where chemotherapy selection was influenced by medical comorbidities. Patients receiving cisplatin-based chemoradiation had an OS rate of 87% at five years, while those treated with carboplatin-doublet or cetuximab-based regimens had lower OS rates of 61% and 47%, respectively. In this context, the upfront TORS treatment might have favorable oncologic outcomes for patients over the age of 70. Additionally, it often eliminates the need for adjuvant therapies, particularly chemotherapy. In fact, Parhar et al. [26] found that 39% of patients were able to avoid all forms of adjuvant therapy, and 74% avoided chemotherapy specifically. Similarly, Costantino et al. [27] analyzed elderly OPSCC patients from the NCDB treated with transoral surgery, categorizing them by risk level. They observed that adjuvant therapy did not improve survival for low- or intermediate-risk patients, while high-risk patients experienced a benefit, though CRT was only slightly superior to RT alone. These results suggest that adjuvant therapy should be reserved primarily for high-risk elderly patients. However, the study’s conclusions are limited by the NCDB’s lack of detailed treatment information (e.g., chemotherapy regimens, adherence), which is especially relevant for elderly populations who often receive “adjusted” therapies. The research did not distinguish between robotic and non-robotic surgeries, further limiting interpretation. Additionally, by focusing on OS, the study may overlook quality of life and functional outcomes, which are particularly important to older patients.

Several studies have examined the functional outcomes following TORS, but there is limited evidence regarding the impact of age on swallowing function after the procedure. Gross et al. [42] found a significant association between increasing age and poorer swallowing outcomes. This was evaluated through speech therapy assessments, the need for gastrostomy tubes, and the Functional Outcome Swallowing Scale (FOSS). Their study focused on patients who underwent TORS for locally advanced oropharyngeal cancer, but they did not define what they considered to be older age. In a more detailed analysis, Dziegielewski et al. [43] identified a significant correlation between patients aged 55 and older and the likelihood of needing a gastrostomy tube after TORS (*p* = 0.047). However, they did not find a significant association between age and the ongoing need for a gastrostomy tube at 12 months post-surgery (*p* = 0.70). Additionally, a study by Philips et al. [28] revealed that although elderly patients were more likely to be discharged on enteric feeds after TORS, the functional outcomes at 3 months, 1 year, and 2 years, measured by enteric feed dependence, nil per os (NPO) status, Functional Oral Intake Scale (FOIS), tracheostomy tube rates, and unplanned readmissions, were comparable to those of non-elderly patients. The necessity for post-operative enteric tube feeds in the elderly may be attributed to poorer baseline function, inadequate swallowing coordination, and a reduced capacity for swallowing therapy.

Notably, studies show that patients with oropharyngeal cancer who undergo definitive nonsurgical treatment may require gastrostomy tube placement in up to 62% of cases [44,45]. Furthermore, the percentage of patients who depend on gastrostomy tubes one year after exclusive chemoradiotherapy (CRT) ranges from 7% to 31% [44,45]. This high rate of dependence can be attributed to the adverse effects of the treatment, such as mucositis and dysphagia. Additionally, the placement of gastrostomy tubes can lead to atrophy of the pharyngeal musculature and cause late esophageal complications, including toxicities and stenosis, which may result in increased long-term reliance on feeding tubes [46]. However, it is important to note that these comparisons are indirect, as elderly-specific prospective head-to-head functional outcome data between TORS and definitive nonsurgical treatment remain scarce.

Overall, data on the safety of transoral surgery for OPSCC in older adults are limited. In NCDB analyses, age was not an independent predictor of postoperative mortality, and length of stay and 30-day readmission rates were comparable between older and younger patients. Older patients, however, were less likely to receive adjuvant RT or CT, even when indicated, likely because of treatment refusal or medical contraindications related to comorbidities. Functional outcomes were not reported, and more granular information on swallowing and quality of life is still lacking [29].

In conclusion, direct comparisons between primary TORS and definitive non-surgical treatment in elderly patients remain limited. Available data suggest broadly comparable oncologic outcomes in selected populations, though interpretation is constrained by retrospective designs and the paucity of functional outcome reporting. Potential advantages of TORS include pathologic risk stratification, reduced long-term gastrostomy dependence, and the opportunity to de-intensify adjuvant therapy. Conversely, surgery carries perioperative risks and may necessitate multimodal treatment in advanced disease. Consequently, treatment selection should be individualized, integrating tumor factors, comorbidity burden, and patient functional status.

### 4.2. TORS for Laryngeal Squamous Cell Carcinoma (LSCC) in Elderly Patients

The use of TORS for supraglottic LSCC in elderly patients is well-supported due to its numerous advantages, which lead to improved surgical, functional, and survival outcomes [47,48]. TOLM has significantly decreased the need for open partial laryngectomies, which is particularly important for elderly patients, as such procedures carry a higher risk of pulmonary complications. While TOLM provides good oncological and functional results, it does have limitations. These include restricted visibility through the laryngoscope, challenges in accessing tumors situated in specific areas, and a steep learning curve for surgeons. In this context, TORS has emerged as a promising approach to overcome these challenges [49]. TORS offers superior visualization of the surgical field compared to TOLM, thanks to its three-dimensional imaging capabilities and the 30° angulation of the optical view. The procedure involves shifting the tongue base horizontally and elevating the laryngopharyngeal tissues while applying anterior traction to the tongue outside the mouth [47,48]. Additionally, the robotic console and instrument design enhance precision, providing a 180° range of motion and reducing physiological tremors. These benefits make TORS a more comfortable and less physically demanding technique for surgeons compared to TOLM and traditional endoscopic methods, which some studies have linked to higher rates of positive surgical margins [47,50]. The technical advantages of TORS may also allow for a less aggressive therapeutic approach, which is crucial for elderly patients [47,50]. Notably, some studies comparing TORS and TOLM suggest that TORS may be associated with lower rates of positive margins. As a result, there is preliminary evidence indicating that TORS could lead to better outcomes in overall survival, disease-free survival, local and regional control, and relapse-free survival [48]. However, these findings should be interpreted with caution, as the current body of evidence is not sufficient to draw definitive conclusions.

A recent review by Lechien [51] examined the functional and therapeutic outcomes of elderly patients undergoing TOLM and TORS for supraglottic LSCC. The findings indicate that age alone does not significantly increase the risk of most postoperative complications for either surgical approach. The only notable exception is aspiration, which appears to be more common in older patients, probably because of age-related sensory mucosal changes. However, careful patient selection and thorough pre- and postoperative swallowing assessments can help reduce this risk. Functional outcomes for elderly patients undergoing TORS showed considerable variation, largely due to differences in tracheotomy practices across institutions. Tracheotomy rates for TORS in laryngeal cancer ranged from 0% to 100%, though in studies where tracheotomy was selectively performed, the rates were significantly lower (0–9%) [52,53]. Decannulation typically occurred within 2 to 11.3 days. Similarly, feeding tube usage varied widely, with reported rates between 44% and 83%. Notably, patients treated with TORS were able to resume an oral diet more quickly (0–12 days) compared to those undergoing TOLM (1.5–14.5 days) [51].

Regarding survival outcomes, a recent systematic review reported 5-year overall survival (OS) rates of 70.1% for TOLM and 78.7% to 80.2% for TORS, with disease-specific survival (DSS) at 82.0% for TOLM and 94.3% for TORS [50]. Although survival rates for elderly patients may be lower compared to younger cohorts, disease-free survival (DFS) remains largely unaffected by age. This suggests that comorbidities and concurrent illnesses, rather than chronological age, are the primary factors influencing overall survival.

### 4.3. The Role of TORS as a De-Escalation Strategy: Can It Be Applied to Elderly Patients?

A significant trend in HNSCC is the implementation of de-escalation therapy. This approach aims to reduce the volume, number of treatment sessions, and radiation dosages to minimize treatment-related toxicity. In cases involving de-escalation treatment, histopathological data obtained from surgical procedures such as TORS can help reduce the adverse side effects associated with non-surgical adjuvant treatments, both in the short and long term [54].

TORS allows for the precise evaluation of intraoperative margins, resulting in a high rate of margin-negative resections and consequently low local recurrence rates. This level of precision opens the possibility of excluding the primary tumor site from the RT field in treating early-stage OPSCC [55,56]. Additionally, it can lead to reduced radiation doses and may even eliminate the need for chemotherapy in treating advanced-stage OPSCC, ultimately decreasing treatment-related toxicity [57].

A 2016 study demonstrated that omitting RT to the primary tumor site in cases of margin-negative, resected T1–T2 p16-positive OPSCC did not significantly impact local disease control. Among 202 patients with T1–T2 disease, 92 did not receive planned RT to the primary tumor bed; 48 of these patients received no adjuvant therapy, while 44 underwent RT limited to the ipsilateral neck. The local recurrence rate in the group that did not receive RT to the primary site was 3%, compared to 0% in those who did. Furthermore, patients who avoided RT to the tumor site experienced better preservation of swallowing function, with only 6.5% requiring a temporary gastrostomy tube, compared to 41% in the group that received RT to the primary site [58].

A Phase II clinical trial (NCT02159703) evaluated the safety and effectiveness of confining radiotherapy to the neck while sparing the primary tumor in 60 patients with stage pT1–pT2, N1–3 p16-positive OPSCC who underwent TORS and selective neck dissection. All participants had favorable pathology at the primary site, with clear surgical margins and no perineural or lymphovascular invasion. The decision for adjuvant RT, with or without chemotherapy, depended on lymph node involvement and the presence of extranodal extension (ENE). The RT target volumes were defined as follows: TV1 included ipsilateral levels II-IV; TV2 covered ipsilateral level V and contralateral levels II-IV; and TV3 addressed areas of ENE. Doses administered were 60 Gy for TV1, 54 Gy for TV2, and 63–66 Gy for TV3, delivered over 30–33 sessions while intentionally excluding the primary tumor site from treatment planning. The study reported a 2-year local control rate of 98.3% and a recurrence-free survival rate of 97.9%. Adverse effects were minimal, with only 3.3% of patients requiring a temporary feeding tube, and a similarly low rate (3.3%) of soft tissue necrosis in the operative bed. However, it is noteworthy that, due to RT planning, the mean unintended dose to the primary tumor bed was 36 Gy, which is comparable to or exceeds doses used in aggressive de-escalation trials such as MC1273 and MC1675.

White et al. [59] conducted a review that included 89 patients, 65% of whom had either T3–T4 tumors or N2–N3 disease. This study found that 92% of the patients underwent TORS as their primary treatment, leading to a two-year OS rate of 89.3%. In another review by Hurtuk et al. [60], 64 patients also received TORS, with 68.4% classified as N2–N3. Their analysis of pathological specimens indicated that 34% of patients with stage III/IV tumors were able to avoid chemoradiotherapy. However, the use of upfront TORS for the treatment of advanced-stage OPSCC should be approached with caution due to the risks associated with a trimodal treatment approach, which can lead to significant short- and long-term toxicities that negatively affect quality of life. For instance, Luckens et al. [61] reported a 28% rate of late soft tissue necrosis in patients treated for advanced OPSCC with TORS followed by adjuvant RT. Nevertheless, recent long-term follow-up data from the ORATOR trial—conducted predominantly in mixed-age populations— found no significant difference in two-year quality-of-life outcomes between treatment strategies, suggesting that in selected cases a trimodal approach may not necessarily result in high long-term toxicity. However, extrapolation of these findings to elderly-specific populations should be undertaken cautiously [39].

Given the potential of TORS to reduce adverse side effects associated with non-surgical adjuvant treatments, it is evident that elderly patients might be the ideal candidates to benefit from this approach. Unfortunately, de-escalation trials have not been comprehensively studied in the HNSCC elderly population. Currently, there are only a small series of published studies assessing the efficacy of hypofractionation specifically in older adults with HNSCC [62,63]. Therefore, future research is warranted to evaluate the role of TORS as a de-escalation strategy in this elderly population.

### 4.4. Factors Influencing Treatment Decisions for Elderly Patients with HNSCC

Selecting the right primary treatment is a key prognostic factor in managing patients with HNSCC. However, older patients often receive inadequate treatment due to their age and existing comorbidities. While the negative impact of comorbidities on prognosis is well-documented [64,65,66], there is limited information on how age affects the quality of treatment for patients with head and neck cancer. For example, sarcopenia (i.e., the age-related, involuntary loss of skeletal muscle mass and strength) is an independent prognostic factor for elderly patients with HNSCC and is associated with chemotherapy-related dose-limiting toxicity [67]. Additionally, cognitive impairment in older HNSCC patients, which may result from cerebrovascular accidents and dementia, is linked to a higher incidence of postoperative delirium in those undergoing surgery [68].

Research indicates that older patients are less likely to undergo intervention with curative intent, with surgery and chemotherapy being offered less frequently. In a study conducted by Hirano et al. [69], which involved 119 patients aged 75 and older, it was found that 26% received substandard treatment. Among these cases, 10.1% were attributed to comorbidities, while 13.4% were linked to patients refusing treatment. Furthermore, the study revealed that the three-year overall survival rate was 18% for the group that received substandard treatment, compared to 77% for those who received standard treatment. However, these results were not categorized by age. Similarly, Derks et al. [70] studied 118 candidates for surgical therapy over the age of 70 and found a substandard treatment rate of 28.8%. In this group, 11% of the cases were due to tumor unresectability, followed by 9.3% related to comorbidities and 5.9% due to patient refusal. Furthermore, Sanabria et al. [71] examined a cohort of 312 patients aged 70 and older and reported a substandard treatment rate of 19.9%. This rate was associated with older age, tumors located in the oro- or hypopharynx, severe comorbidities, advanced clinical stage, and low performance status. Importantly, those in the substandard treatment group experienced reduced overall survival and cancer-specific survival rates.

The data shows that selecting substandard treatments based on a patient’s age, tumor location, or mild to moderate comorbidities can negatively impact their prognosis. Surgeons often perceive older patients as less capable of enduring major procedures and more likely to face postoperative complications. However, this belief is not well-supported by medical literature. Additionally, there is a common notion that for older patients, preserving quality of life is more important than extending lifespan. As a result, physicians may recommend less aggressive treatments, which can increase the risk of disease recurrence.

Patients and their families might also refuse standard treatments due to widespread misconceptions about the resilience of older individuals or the influence of surgeons on their choices [70].

However, when carefully assessed, older patients with cancer express strong preferences for receiving information and participating in decisions about their care [72]. Best oncological practice dictates that a framework for clinical decision-making in older adults with cancer is built on a foundation of evidence-based medicine. A principled approach to decision-making, using a framework that includes a geriatric assessment [73,74,75,76,77] for oncologic patients [78], will minimize under- and overtreatment while optimizing decisions, matching treatment selection with age-related vulnerability, and aligning expected outcomes with patient preferences [79].

## 5. Conclusions

TORS has emerged as a promising treatment option for elderly patients with HNSCC, particularly for those with oropharyngeal and laryngeal cancers. Available studies suggest that, in carefully selected older adults, TORS can achieve oncologic outcomes comparable to those observed in younger populations. However, these findings derive largely from retrospective cohorts and should be interpreted with caution. While transoral surgery may reduce the need for treatment intensification and thereby limit therapy-related toxicity in selected cases, robust elderly-specific comparative data—particularly regarding functional and quality-of-life outcomes—remain limited. Evidence suggesting functional advantages over definitive CRT is largely indirect or extrapolated from mixed-age prospective trials. Moreover, elderly patients continue to be underrepresented in clinical studies, and standardized treatment algorithms tailored to this population are lacking. Although de-escalation strategies incorporating TORS show potential to reduce long-term morbidity, further prospective investigation is required to validate their role specifically in aging populations. Future studies should prioritize refining patient selection criteria, optimizing perioperative care, and assessing long-term functional and oncologic outcomes in elderly patients undergoing TORS. A multidisciplinary approach, incorporating geriatric assessments, preoperative speech therapy evaluation of swallowing function and coordination, and personalized treatment plans, is crucial for enhancing outcomes and quality of life for older adults with HNSCC.

## Data Availability

No new data were created or analyzed in this study. Data sharing does not apply to this article.

## References

[1] Fitzmaurice C., Abate D., Abbasi N., Abbastabar H., Abd-Allah F., Abdel-Rahman O., Abdelalim A., Abdoli A., Abdollahpour I., Global Burden of Disease Cancer Collaboration (2019). Global, Regional, and National Cancer Incidence, Mortality, Years of Life Lost, Years Lived with Disability, and Disability-Adjusted Life-Years for 29 Cancer Groups, 1990 to 2017: A Systematic Analysis for the Global Burden of Disease Study. JAMA Oncol..

[2] Sikora A.G., Toniolo P., DeLacure M.D. (2004). The changing demographics of head and neck squamous cell carcinoma in the United States. Laryngoscope.

[3] Gillison M.L., Broutian T., Pickard R.K.L., Tong Z.Y., Xiao W., Kahle L., Graubard B.I., Chaturvedi A.K. (2012). Prevalence of oral HPV infection in the United States, 2009–2010. JAMA.

[4] Zumsteg Z.S., Cook-Wiens G., Yoshida E., Shiao S.L., Lee N.Y., Mita A., Jeon C., Goodman M.T., Ho A.S. (2016). Incidence of Oropharyngeal Cancer Among Elderly Patients in the United States. JAMA Oncol..

[5] Windon M.J., D’Souza G., Rettig E.M., Westra W.H., van Zante A., Wang S.J., Ryan W.R., Mydlarz W.K., Ha P.K., Miles B.A. (2018). Increasing prevalence of human papillomavirus-positive oropharyngeal cancers among older adults. Cancer.

[6] Rettig E.M., Fakhry C., Khararjian A., Westra W.H. (2018). Age Profile of Patients with Oropharyngeal Squamous Cell Carcinoma. JAMA Otolaryngol. Head Neck Surg..

[7] Fentiman I.S., Tirelli U., Monfardini S., Schneider M., Festen J., Cognetti F., Aapro M.S. (1990). Cancer in the elderly: Why so badly treated?. Lancet.

[8] Daly M.E., Lau D.H., Farwell D.G., Luu Q., Donald P.J., Chen A.M. (2013). Feasibility and toxicity of concurrent chemoradiation for elderly patients with head and neck cancer. Am. J. Otolaryngol..

[9] Reid B.C., Alberg A.J., Klassen A.C., Samet J.M., Rozier R.G., Garcia I., Winn D.M. (2001). Comorbidity and survival of elderly head and neck carcinoma patients. Cancer.

[10] Haehl E., Rühle A., David H., Kalckreuth T., Sprave T., Stoian R., Becker C., Knopf A., Grosu A.L., Nicolay N.H. (2020). Radiotherapy for geriatric head-and-neck cancer patients: What is the value of standard treatment in the elderly?. Radiat. Oncol..

[11] Tobias J.S., Monson K., Gupta N., Macdougall H., Glaholm J., Hutchison I., Kadalayil L., Hackshaw A., UK Head and Neck Cancer Trialists’ Group (2010). Chemoradiotherapy for locally advanced head and neck cancer: 10-year follow-up of the UK Head and Neck (UKHAN1) trial. Lancet Oncol..

[12] Machtay M., Moughan J., Trotti A., Garden A.S., Weber R.S., Cooper J.S., Forastiere A., Ang K.K. (2008). Factors associated with severe late toxicity after concurrent chemoradiation for locally advanced head and neck cancer: An RTOG analysis. J. Clin. Oncol. Off. J. Am. Soc. Clin. Oncol..

[13] Pignon J.P., le Maître A., Maillard E., Bourhis J., MACH-NC Collaborative Group (2009). Meta-analysis of chemotherapy in head and neck cancer (MACH-NC): An update on 93 randomised trials and 17,346 patients. Radiother. Oncol. J. Eur. Soc. Ther. Radiol. Oncol..

[14] Bonner J.A., Harari P.M., Giralt J., Cohen R.B., Jones C.U., Sur R.K., Raben D., Baselga J., Spencer S.A., Zhu J. (2010). Radiotherapy plus cetuximab for locoregionally advanced head and neck cancer: 5-year survival data from a phase 3 randomised trial, and relation between cetuximab-induced rash and survival. Lancet Oncol..

[15] Handforth C., Clegg A., Young C., Simpkins S., Seymour M.T., Selby P.J., Young J. (2015). The prevalence and outcomes of frailty in older cancer patients: A systematic review. Ann. Oncol..

[16] de Almeida J.R., Li R., Magnuson J.S., Smith R.V., Moore E., Lawson G., Remacle M., Ganly I., Kraus D.H., Teng M.S. (2015). Oncologic Outcomes After Transoral Robotic Surgery: A Multi-institutional Study. JAMA Otolaryngol. Head Neck Surg..

[17] de Almeida J.R., Byrd J.K., Wu R., Stucken C.L., Duvvuri U., Goldstein D.P., Miles B.A., Teng M.S., Gupta V., Genden E.M. (2014). A systematic review of transoral robotic surgery and radiotherapy for early oropharynx cancer: A systematic review. Laryngoscope.

[18] Weinstein G.S., O’Malley B.W., Magnuson J.S., Carroll W.R., Olsen K.D., Daio L., Moore E.J., Holsinger F.C. (2012). Transoral robotic surgery: A multicenter study to assess feasibility, safety, and surgical margins. Laryngoscope.

[19] Weinstein G.S., Quon H., Newman H.J., Chalian J.A., Malloy K., Lin A., Desai A., Livolsi V.A., Montone K.T., Cohen K.R. (2012). Transoral robotic surgery alone for oropharyngeal cancer: An analysis of local control. Arch. Otolaryngol. Head Neck Surg..

[20] Weinstein G.S., O’Malley B.W., Cohen M.A., Quon H. (2010). Transoral robotic surgery for advanced oropharyngeal carcinoma. Arch. Otolaryngol. Head Neck Surg..

[21] Mydlarz W.K., Chan J.Y., Richmon J.D. (2015). The role of surgery for HPV-associated head and neck cancer. Oral Oncol..

[22] De Virgilio A., Iocca O., Malvezzi L., Di Maio P., Pellini R., Ferreli F., Cugini G., Colombo G., Spriano G. (2019). The Emerging Role of Robotic Surgery among Minimally Invasive Surgical Approaches in the Treatment of Hypopharyngeal Carcinoma: Systematic Review and Meta-Analysis. J. Clin. Med..

[23] Smith R.V. (2014). Transoral robotic surgery for larynx cancer. Otolaryngol. Clin. N. Am..

[24] Doazan M., Hans S., Morinière S., Lallemant B., Vergez S., Aubry K., De Monès E., Espitalier F., Jegoux F., Pradat P. (2018). Oncologic outcomes with transoral robotic surgery for supraglottic squamous cell carcinoma: Results of the French Robotic Surgery Group of GETTEC. Head Neck.

[25] Jackson R.S., Chen S., Last A., Khan A., Kallogjeri D., Van Abel K.M., Moore E.J., Pipkorn P. (2019). Multi-institutional analysis of outcomes following transoral surgery for HPV-positive oropharyngeal squamous cell carcinoma in elderly patients. Head Neck.

[26] Parhar H.S., Shimunov D., Newman J.G., Cannady S.B., Rajasekaran K., O’Malley B.W., Chalian A.A., Rassekh C.H., Cohen R.B., Lin A. (2020). Oncologic Outcomes Following Transoral Robotic Surgery for Human Papillomavirus-Associated Oropharyngeal Carcinoma in Older Patients. JAMA Otolaryngol. Head Neck Surg..

[27] Costantino A., Sampieri C., Haughey B.H., Alamoudi U., De Virgilio A., Magnuson J.S. (2025). Adjuvant treatment in elderly patients undergoing transoral surgery for HPV-related oropharyngeal cancer. Oral Oncol..

[28] Philips R., Topf M.C., Crawley M.B., Swendseid B., Luginbuhl A., Curry J., Cognetti D. (2020). Functional and survival outcomes in elderly patients undergoing transoral robotic surgery. Oral Oncol..

[29] Costantino A., Haughey B., Alamoudi U., Biskup M., Magnuson J.S. (2024). Safety and Postoperative Outcomes of Transoral Surgery for Oropharyngeal Carcinoma in Older Adults. JAMA Otolaryngol. Head Neck Surg..

[30] Costantino A., Haughey B., Zhu J., Alamoudi U., Magnuson J.S. (2025). Transoral Surgery Versus Radiotherapy as Primary Treatment for HPV-Related Oropharyngeal Cancer in the Elderly. Head Neck.

[31] Costantino A., Haughey B., Alamoudi U., Magnuson J.S. (2024). Challenges in treating oropharyngeal cancer in the elderly: The role of transoral surgery. Oral Oncol..

[32] Motz K., Herbert R.J., Fakhry C., Quon H., Kang H., Kiess A.P., Eisele D.W., Koch W.M., Frick K.D., Gourin C.G. (2018). Short- and long-term outcomes of oropharyngeal cancer care in the elderly. Laryngoscope.

[33] Sinha P., Haughey B.H., Kallogjeri D., Jackson R.S. (2019). Long-term analysis of transorally resected p16+ Oropharynx cancer: Outcomes and prognostic factors. Laryngoscope.

[34] Moore E.J., Van Abel K.M., Price D.L., Lohse C.M., Olsen K.D., Jackson R.S., Martin E.J. (2018). Transoral robotic surgery for oropharyngeal carcinoma: Surgical margins and oncologic outcomes. Head Neck.

[35] Dale O.T., Sood S., Shah K.A., Han C., Rapozo D., Mehanna H., Winter S.C. (2016). Long-term survival outcomes in patients with surgically treated oropharyngeal cancer and defined human papilloma virus status. J. Laryngol. Otol..

[36] Hoffmann M., Quabius E.S., Tribius S., Gebhardt S., Görögh T., Hedderich J., Huber K., Dunst J., Ambrosch P. (2018). Influence of HPV-status on survival of patients with tonsillar carcinomas (TSCC) treated by CO2-laser surgery plus risk adapted therapy—A 10 year retrospective single centre study. Cancer Lett..

[37] Parhar H.S., Gausden E., Patel J., Prisman E., Anderson D.W., Durham J.S., Rush B. (2018). Analysis of readmissions after transoral robotic surgery for oropharyngeal squamous cell carcinoma. Head Neck.

[38] Stokes W., Ramadan J., Lawson G., Ferris F.R.L., Holsinger F.C., Turner M.T. (2021). Bleeding Complications After Transoral Robotic Surgery: A Meta-Analysis and Systematic Review. Laryngoscope.

[39] Nichols A.C., Theurer J., Prisman E., Read N., Berthelet E., Tran E., Fung K., de Almeida J.R., Bayley A., Goldstein D.P. (2022). Randomized Trial of Radiotherapy Versus Transoral Robotic Surgery for Oropharyngeal Squamous Cell Carcinoma: Long-Term Results of the ORATOR Trial. J. Clin. Oncol..

[40] Hanasoge S., Magliocca K.R., Switchenko J.M., Saba N.F., Wadsworth J.T., El-Deiry M.W., Shin D.M., Khuri F., Beitler J.J., Higgins K.A. (2016). Clinical outcomes in elderly patients with human papillomavirus-positive squamous cell carcinoma of the oropharynx treated with definitive chemoradiation therapy. Head Neck.

[41] Zumsteg Z.S., Lok B.H., Ho A.S., Drill E., Zhang Z., Riaz N., Shiao S.L., Ma J., McBride S.M., Tsai C.J. (2017). The toxicity and efficacy of concomitant chemoradiotherapy in patients aged 70 years and older with oropharyngeal carcinoma in the intensity-modulated radiotherapy era. Cancer.

[42] Gross J.H., Townsend M., Hong H.Y., Miller E., Kallogjeri D., Zenga J., Pipkorn P., Jackson R.S., Haughey B., Rich J.T. (2020). Predictors of swallow function after transoral surgery for locally advanced oropharyngeal cancer. Laryngoscope.

[43] Dziegielewski P.T., Teknos T.N., Durmus K., Old M., Agrawal A., Kakarala K., Marcinow A., Ozer E. (2013). Transoral robotic surgery for oropharyngeal cancer: Long-term quality of life and functional outcomes. JAMA Otolaryngol. Head Neck Surg..

[44] Setton J., Lee N.Y., Riaz N., Huang S.H., Waldron J., O’Sullivan B., Zhang Z., Shi W., Rosenthal D.I., Hutcheson K.A. (2015). A multi-institution pooled analysis of gastrostomy tube dependence in patients with oropharyngeal cancer treated with definitive intensity-modulated radiotherapy. Cancer.

[45] Shiley S.G., Hargunani C.A., Skoner J.M., Holland J.M., Wax M.K. (2006). Swallowing function after chemoradiation for advanced stage oropharyngeal cancer. Otolaryngol. Head Neck Surg. Off. J. Am. Acad. Otolaryngol..

[46] Chen A.M., Li B.Q., Jennelle R.L., Lau D.H., Yang C.C., Courquin J., Vijayakumar S., Purdy J.A. (2010). Late esophageal toxicity after radiation therapy for head and neck cancer. Head Neck.

[47] Lechien J.R., Fakhry N., Saussez S., Chiesa-Estomba C.M., Chekkoury-Idrissi Y., Cammaroto G., Melkane A.E., Barillari M.R., Crevier-Buchman L., Ayad T. (2020). Surgical, clinical and functional outcomes of transoral robotic surgery for supraglottic laryngeal cancers: A systematic review. Oral Oncol..

[48] Loubieres C., Hans S., Lechien J.R., Ansarin M., Atallah S., Barbut J., Bizeau A., Burkey B., Céruse P., Choussy O. (2025). Expert perspectives for transoral robotic versus laser surgery for supraglottic carcinomas. Eur. Arch. Oto-Rhino-Laryngol..

[49] Hans S., Baudouin R., Circiu M.P., Couineau F., Lisan Q., Crevier-Buchman L., Lechien J.R. (2022). Laryngeal Cancer Surgery: History and Current Indications of Transoral Laser Microsurgery and Transoral Robotic Surgery. J. Clin. Med..

[50] Lechien J.R., Hans S. (2024). Survival, Surgical, and functional outcomes of transoral laser microsurgery for cT1-T3 supraglottic laryngeal Cancers: A systematic review. Oral Oncol..

[51] Lechien J.R. (2024). Transoral Laser Microsurgery and Transoral Robotic Surgery in Aging Patients: A State-of-The-Art Review. Clin. Interv. Aging.

[52] Lallemant B., Chambon G., Garrel R., Kacha S., Rupp D., Galy-Bernadoy C., Chapuis H., Lallemant J.G., Pham H.T. (2013). Transoral robotic surgery for the treatment of T1-T2 carcinoma of the larynx: Preliminary study. Laryngoscope.

[53] Karabulut B., Deveci I., Sürmeli M., Şahin-Yilmaz A., Oysu Ç. (2018). Comparison of functional and oncological treatment outcomes after transoral robotic surgery and open surgery for supraglottic laryngeal cancer. J. Laryngol. Otol..

[54] O’Hara J., Warner L., Fox H., Hamilton D., Meikle D., Counter P., Robson A., Goranova R., Iqbal S., Kelly C. (2021). Primary transoral robotic surgery +/− adjuvant therapy for oropharyngeal squamous cell carcinoma—A large observational single-centre series from the United Kingdom. Clin. Otolaryngol..

[55] van Loon J.W., Smeele L.E., Hilgers F.J., van den Brekel M.W. (2015). Outcome of transoral robotic surgery for stage I–II oropharyngeal cancer. Eur. Arch. Oto-Rhino-Laryngol..

[56] Molteni G., Bassani S., Arsie A.E., Zampieri E., Mannelli G., Orlandi E., Bossi P., De Virgilio A. (2024). Role of TORS as De-Escalation Strategy in HPV-Related Oropharyngeal Cancer, What We Need to Know. Healthcare.

[57] Park Y.M., Kim H.R., Cho B.C., Keum K.C., Cho N.H., Kim S.H. (2017). Transoral robotic surgery-based therapy in patients with stage III-IV oropharyngeal squamous cell carcinoma. Oral Oncol..

[58] Sinha P., Pipkorn P., Thorstad W.L., Gay H.A., Haughey B.H. (2016). Does elimination of planned postoperative radiation to the primary bed in p16-positive, transorally-resected oropharyngeal carcinoma associate with poorer outcomes?. Oral Oncol..

[59] White H.N., Moore E.J., Rosenthal E.L., Carroll W.R., Olsen K.D., Desmond R.A., Magnuson J.S. (2010). Transoral robotic-assisted surgery for head and neck squamous cell carcinoma: One- and 2-year survival analysis. Arch. Otolaryngol. Head Neck Surg..

[60] Hurtuk A., Agrawal A., Old M., Teknos T.N., Ozer E. (2011). Outcomes of transoral robotic surgery: A preliminary clinical experience. Otolaryngol. Head Neck Surg..

[61] Lukens J.N., Lin A., Gamerman V., Mitra N., Grover S., McMenamin E.M., Weinstein G.S., O’Malley B.W., Cohen R.B., Orisamolu A. (2014). Late consequential surgical bed soft tissue necrosis in advanced oropharyngeal squamous cell carcinomas treated with transoral robotic surgery and postoperative radiation therapy. Int. J. Radiat. Oncol. Biol. Phys..

[62] Bledsoe T.J., Noble A.R., Reddy C.A., Burkey B.B., Greskovich J.F., Nwizu T., Adelstein D.J., Saxton J.P., Koyfman S.A. (2016). Split-Course Accelerated Hypofractionated Radiotherapy (SCAHRT): A Safe and Effective Option for Head and Neck Cancer in the Elderly or Infirm. Anticancer Res..

[63] De Felice F., Vetrone L., Bulzonetti N., Caiazzo R., Marampon F., Musio D., Tombolini V. (2019). Hypofractionated radiotherapy combined with cetuximab in vulnerable elderly patients with locally advanced head and neck squamous cell carcinoma. Med. Oncol..

[64] Piccirillo J.F. (2000). Importance of comorbidity in head and neck cancer. Laryngoscope.

[65] de Cássia Braga Ribeiro K., Kowalski L.P., Latorre M.D.R. (2003). Perioperative complications, comorbidities, and survival in oral or oropharyngeal cancer. Arch. Otolaryngol. Head Neck Surg..

[66] Gourin C.G., McAfee W.J., Neyman K.M., Howington J.W., Podolsky R.H., Terris D.J. (2005). Effect of comorbidity on quality of life and treatment selection in patients with squamous cell carcinoma of the head and neck. Laryngoscope.

[67] Chargi N., Bril S.I., Emmelot-Vonk M.H., de Bree R. (2019). Sarcopenia is a prognostic factor for overall survival in elderly patients with head-and-neck cancer. Eur. Arch. Oto-Rhino-Laryngol..

[68] van Deudekom F.J., Schimberg A.S., Kallenberg M.H., Slingerland M., van der Velden L.A., Mooijaart S.P. (2017). Functional and cognitive impairment, social environment, frailty and adverse health outcomes in older patients with head and neck cancer, a systematic review. Oral Oncol..

[69] Hirano M., Mori K. (1998). Management of cancer in the elderly: Therapeutic dilemmas. Otolaryngol. Head Neck Surg..

[70] Derks W., De Leeuw J.R., Hordijk G.J., Winnubst J.A. (2003). Elderly patients with head and neck cancer: Short-term effects of surgical treatment on quality of life. Clin. Otolaryngol. Allied Sci..

[71] Sanabria A., Carvalho A.L., Vartanian J.G., Magrin J., Ikeda M.K., Kowalski L.P. (2007). Factors that influence treatment decision in older patients with resectable head and neck cancer. Laryngoscope.

[72] Paillaud E., Canoui-Poitrine F., Varnier G., Anfasi-Ebadi N., Guery E., Saint-Jean O., Gisselbrecht M., Aparicio T., Bastuji-Garin S., Laurent M. (2017). Preferences about information and decision-making among older patients with and without cancer. Age Ageing.

[73] Charlson M.E., Pompei P., Ales K.L., MacKenzie C.R. (1987). A new method of classifying prognostic comorbidity in longitudinal studies: Development and validation. J. Chronic Dis..

[74] Piccirillo J.F., Wells C.K., Sasaki C.T., Feinstein A.R. (1994). New clinical severity staging system for cancer of the larynx. Five-year survival rates. Ann. Otol. Rhinol. Laryngol..

[75] Kaplan M.H., Feinstein A.R. (1974). The importance of classifying initial co-morbidity in evaluating the outcome of diabetes mellitus. J. Chronic Dis..

[76] Bellera C.A., Rainfray M., Mathoulin-Pélissier S., Mertens C., Delva F., Fonck M., Soubeyran P.L. (2012). Screening older cancer patients: First evaluation of the G-8 geriatric screening tool. Ann. Oncol..

[77] Overcash J., Ford N., Kress E., Ubbing C., Williams N. (2019). Comprehensive Geriatric Assessment as a Versatile Tool to Enhance the Care of the Older Person Diagnosed with Cancer. Geriatrics.

[78] Mohile S.G., Dale W., Somerfield M.R., Schonberg M.A., Boyd C.M., Burhenn P.S., Canin B., Cohen H.J., Holmes H.M., Hopkins J.O. (2018). Practical Assessment and Management of Vulnerabilities in Older Patients Receiving Chemotherapy: ASCO Guideline for Geriatric Oncology. J. Clin. Oncol..

[79] DuMontier C., Loh K.P., Soto-Perez-de-Celis E., Dale W. (2021). Decision Making in Older Adults with Cancer. J. Clin. Oncol..

